# Apport de la biopsie radioguidée dans le diagnostic histopathologique des tumeurs de l'enfant: expérience de l'Hôpital d'Enfant de Rabat

**DOI:** 10.11604/pamj.2015.21.318.5657

**Published:** 2015-08-28

**Authors:** Mohamed Réda El Ochi, Salma Bellarbi, Lamiae Rouas, Najat Lamalmi, Abderrahmane Malihy, Zaitouna Alhamany, Nadia Cherradi

**Affiliations:** 1 Laboratoire d'Anatomie et de Cytologie Pathologique, Hôpital d'Enfant, Rabat, Maroc

**Keywords:** Biopsie, radioguidée, tumeur, enfant, biopsy, radio-guided, tumor, infant

## Abstract

La biopsie radioguidée constitue une alternative à la biopsie chirurgicale invasive et à la cytologie pour le diagnostic des tumeurs pédiatriques. L'intérêt de notre étude est d’évaluer la valeur diagnostique des biopsies radioguidées examinées au laboratoire d'anatomopathologie de l'hôpital d'Enfants de Rabat (HER). L’étude a porté sur 78 biopsies radioguidées recueillies dans notre laboratoire entre janvier 2008 et décembre 2011. l’âge moyen des patients était de 5 ans et 10 mois avec une prédominance masculine (65,4%). La tumeur était abdominale dans 80% des cas, thoracique dans 15% cas, thoracique et abdominale dans 2,5% et sacrée dans 1,2%. Les biopsies étaient écho-guidées dans 90% des cas et scannoguidées dans 10% des cas. Le diagnostic histopathologique était posé dans 89% des cas. L'immuno-histochimie a été indiquée dans 35% des cas. Les diagnostics les plus fréquents étaient: tumeurs neuroblastiques (42 cas), lymphomes non hodgkiniens (10 cas), rhabdomyosarcomes (6 cas), autres (sarcome d'Ewing, néphroblastomes, tumeur myofibroblastique inflammatoire, maladies de Hodgkin, leucémie aiguë, hépatoblastome et ostéosarcome). Dans notre série, la biopsie radioguidée a permis un diagnostic histopathologique certain dans 89% des cas. Elle nécessite une étroite collaboration entre clinicien, radiologue et anatomopathologiste pour discuter son indication, afin de diminuer le nombre de biopsies peu ou non représentatives.

## Introduction

Les tumeurs de l'enfant sont dominées par les tumeurs du blastème, les lymphomes et les sarcomes. Ces derniers peuvent se présenter sous forme de tumeurs à petites cellules rondes [1]. La biopsie radioguidée percutanée joue un rôle de plus en plus croissant dans le diagnostic et la prise en charge. Elle permet d'obtenir un matériel adéquat pour l’étude histopathologique, immunohistochimique, cytogénétique et de biologie moléculaire [1, 2]. Cet examen est plus performant que la cytologie [2] et constitue une alternative à la biopsie chirurgicale invasive. L'intérêt de notre étude est d’évaluer la valeur diagnostique des biopsies radioguidées observées au Laboratoire d'Anatomopathologie de l'Hôpital d'Enfants de Rabat (HER).

## Méthodes

L’étude a porté sur l'exploitation de 78 fiches anatomopathologiques de biopsies radioguidées prises en charge au Laboratoire d'Anatomopathologie de l'HER durant la période de janvier 2008 à décembre 2011. Les données étudiées étaient les suivantes: l’âge, le sexe, la localisation de la lésion, le diagnostic morphologique et le diagnostic final après immunomarquage.

## Résultats

**Figure 1:**
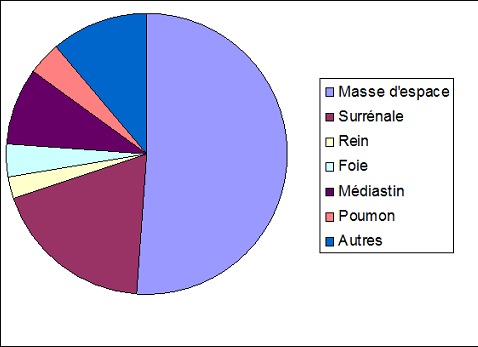
Siège des biopsies

Les principales données cliniques sont répertoriées dans le Tableau 1. L’âge moyen de nos patients était de 5 ans et 10 mois (3 mois à 15 ans) avec une prédominance masculine (65,4% de garçons- 34,6% de filles). La biopsie a été réalisée dans 93,5% des cas pour tumeur primitive, dans 5% des cas pour métastases et dans 1,5% des cas pour récidive tumorale (Tableau 2). Le siège de la biopsie était comme suit: (Figure 1): abdominal dans 63 cas (80%): masse d'espace (52,5%), surrénalien (19,2%), rénal (2,5%), hépatique (3,8%), hépatosplénique et rectal (1,2% chacun); thoracique dans 12 cas (15%): Médiastinal (9%), pulmonaire (3,8%) et médiastinopulmonaire (2,5%); thoracique et abdominal dans 2 cas (2,5%); sacré dans 1cas (1,2%). Les biopsies étaient échoguidées dans 70 cas (90%) et scannoguidées dans 8 cas (10%). Le diagnostic histologique a été posé dans 89% des cas avec recours à un complément immunohistochimique dans 35 cas (45%). Les types histologiques retrouvés étaient par ordre de fréquence décroissant (Tableau 1): tumeurs neuroblastiques (53,8%), lymphomes non hodgkiniens (12,8%), rhabdomyosarcomes (7,7%), sarcome d'Ewing (3,8%), néphroblastomes (2,5%), tumeur myofibroblastique inflammatoire (2,5%), lymphome de Hodgkin (2,5%) et leucémie aigue, ostéosarcome et hépatoblastome (1,2% chacun). Dans 6 cas, le diagnostic histologique n’était pas possible du fait de l'exiguïté du prélèvement et/ou d'un immunomarquage non contributif (4 cas de tumeur abdominal et 2 cas de tumeur médiastinale). Dans 2 cas de tumeur abdominale d'espace, le prélèvement n’était pas tumoral.

**Tableau 1:** Principales données cliniques

Moyenne d’âge au moment du diagnostic	5 ans et 10 mois (patients âgés entre 3 mois et 15 ans)
Sex ratio masculin/Féminin	51/27
**Diagnostic**	
Tumeurs neuroblastiques	42 cas
Lymphomes non hodgkiniens	10 cas
Rhabdomyosarcome	6 cas
Néphroblastome	2 cas
Sarcome d'Ewing	3 cas
Leucémie aigue	1 cas
Hépatoblastome	1 cas
TMI	2 cas
Ostéosarcome	1 cas
Maladie de Hodgkin	2 cas
Non concluant	6 cas

**Tableau 2:** Circonstances de diagnostic

**Tumeur primitive**		73 cas
**Métastases**	- Hépatiques (sarcomed'Ewing et neuroblastome)	2 cas
	- Pulmonaires (sarcome d'Ewing et néphroblastome)	2 cas
Récidive (maladie de hodgkin médiastinale)		1 cas

## Discussion

En pathologie tumorale pédiatrique et malgré les avancées de l'imagerie médicale, il est important d'avoir un diagnostic histologique pré-thérapeutique [3]. Chez l'enfant, on peut avoir recours à la cytoponction, à la biopsie chirurgicale ou bien à la biopsie percutanée radioguidée [3]. La cytoponction a été proposée comme méthode de choix dans le diagnostic des tumeurs solides de l'enfant. Cependant, les tumeurs à cellules rondes, peuvent avoir un aspect cytologique similaire nécessitant alors une vérification histologique [3]. La biopsie radioguidée est un moyen diagnostique très utile dans le diagnostic des tumeurs malignes de l'enfant [2] et moins invasif que la biopsie chirurgicale. Elle est de plus en plus pratiquée en oncologie pédiatrique notamment pour la prise en charge des tumeurs solides et des lymphomes [4]. Elle est d'un apport majeur dans les tumeurs difficiles à réséquer et celles qui peuvent bénéficier d'une chimiothérapie néo adjuvante. Le prélèvement biopsique obtenu permet généralement de réaliser l’étude histopathologique, immunohistochimique et moléculaire. Les séries cliniques publiées sur les biopsies radioguidées ont montré un taux de précision diagnostique de 65 à 100% [4].

**Description de la biopsie radioguidée:** la technique de biopsie: elle est la même pour de nombreux sites avec quelques particularités pour les tumeurs hépatiques et pulmonaires [4]. Le plus souvent, on a recours à l'anesthésie générale ou à la sédation [4, 5] pour contrôler l'anxiété et les mouvements de l'enfant. Après la réalisation d'une incision cutanée de 2 à 3 mm, le trocart est dirigé sous contrôle radiologique vers la lésion. Le doppler couleur permet d'identifier et d’éviter les vaisseaux sanguins [4]. On utilise des aiguilles de 22 gauge [6].

*Le choix entre l’échographie et le scanner:* il est préférable d'utiliser l’échographie pour les lésions qui peuvent être visualisées par cette technique [1, 4]. Couplée au doppler, elle permet d’éviter les lésions vasculaires accidentelles [3], l’étude de la vascularisation de la tumeur et le choix de zones viables à prélever [3]. Les lésions peu visibles à l’échographie, les lésions pulmonaires et les lésions osseuses de la colonne vertébrale et du pelvis sont mieux approchées par le scanner [1, 4].

*Taille et nombre des fragments biopsiques:* l’étude morphologique de routine et de biologie moléculaire nécessite une taille adéquate [7]. Celle-ci n'est pas prédéterminée pour tous les types de tumeurs. Elle n'est pas cruciale pour les tumeurs uniformes, à l'inverse des tumeurs hétérogènes qui nécessitent la réalisation de biopsies multiples dans différents sites [7, 8]. D'autres tumeurs comme les lymphomes, où l'architecture de la prolifération joue un rôle diagnostique important, nécessitent des biopsies relativement larges [8].

*Résultats:* la biopsie radioguidée permet d'obtenir un diagnostic histopathologique dans 65 à 100% des cas selon les séries [5]. En général, les résultats sont significatifs pour les tumeurs primitives que les récidives, pour les tumeurs malignes que bénignes et pour les lésions larges que petites [4].

*Causes d’échec de la biopsie radioguidée:* l’échec est lié soit au geste biopsique qui ne ramène pas le tissu tumoral approprié, soit à un échantillonnage non représentatif [7]. Ceci peut être réduit par la réalisation de biopsies larges et multiples intéressant différents sites de la tumeur et évitant les zones de nécrose [7].

*Avantages par rapport à la biopsie chirurgicale et à la cytologie:* la biopsie radioguidée est de réalisation plus facile et plus rapide, moins invasive, entraine moins de morbidité et permet de démarrer le traitement rapidement sans suites postopératoires [7, 9]. Dans une étude brésilienne ayant portée sur 101 biopsies scanno-guidées, les résultats étaient similaires à la biopsie chirurgicale avec un diagnostic histologique dans 76,6% et 90,8% des cas respectivement (.05 < P <.10) [6]. Quant à la cytologie, elle est souvent peu utile pour le diagnostic des tumeurs de l'enfant [4]. Plusieurs types de tumeurs, en particulier les tumeurs à cellules rondes de l'enfant peuvent avoir le même aspect cytologique [3]. Les travaux rapportés ont conclu que la biopsie radioguidée a une valeur diagnostique supérieure à la cytoponction avec une sensibilité de 95% et 77% respectivement [10].

*Complications:* la biopsie radioguidée est moins utilisée chez l'enfant que chez l'adulte [3]. Ceci est du au risque de traumatiser accidentellement un organe vital, d'entrainer des hémorragies et des infections chez des enfants déjà altérés ou une diffusion de la tumeur le long du trajet biopsique [7]. Ces complications sont devenues très rares avec le développement des techniques radiologiques. L'essaimage de la tumeur à travers le trajet de la biopsie est une complication connue de la cytoponction et de la biopsie percutanée [3]. Sa fréquence va de 1/625 à 1/8500 cas [3]. Deux cas d'essaimage tumoral ont été rapportés dans la population pédiatrique chez des patients suivis pour néphroblastomes [3].

### Apport de la biopsie radioguidée Selon la pathologie et par ordre de fréquence observée dans notre série

*Le neuroblastome:* 42 cas ont été diagnostiqués dans notre série soit 54% des cas. Le neuroblastome est la tumeur solide extra-craniale maligne la plus fréquente de l'enfant [11]. Il représente 8% des cancers de l'enfant et 15% de causes de décès par cancer dans cette tranche d’âge. C'est une tumeur qui dérive des cellules neuroectodermiques primitives du système nerveux sympathique. Les modalités évolutives sont diverses allant de la rémission spontanée au la diffusion métastatique précoce [11]. Le traitement dépend de l’âge du patient, de l'extension de la maladie et des facteurs histologiques et génétiques. La biopsie chirurgicale a été le pilier du diagnostic définitif du neuroblastome [11]. La biopsie radioguidée a été proposée comme une alternative moins invasive. Le tissu obtenu permet non seulement l’étude morphologique mais également l'analyse des facteurs pronostiques comme le N-myc et la ploïdie [2]. Du fait de l'hétérogénéité de cette tumeur, le sous type histologique ne peut être déterminé avec précision sur une biopsie. Ceci n'est pas un problème majeur du fait que l'amplification du N-myc, la délétion du bras court du chromosome 1 et les anomalies du bras long du chromosome 17 ont un impact sur le pronostic qui dépasse de loin la caractérisation du sous type histologique dans la majorité des cas [2]. Une étude récente de Hassan et coll. a démontré qu'il n'y a pas de différence significative entre la biopsie percutanée et la biopsie chirurgicale. Cependant, cette dernière a été plus fréquemment associée à des complications majeures [11].

*Les lymphomes:* 10 cas ont été diagnostiqués dans notre série soit 13% des cas. Les lymphomes non hodgkiniens représentent environ 10-15% [12]. Ils diffèrent des lymphomes de l'adulte car ils sont presque tous de haut grade histologique de malignité, ont une architecture diffuse et une atteinte fréquente extra-ganglionnaire. Trois groupes histologiques prédominent chez l'enfant: le lymphome de Burkitt de topographie essentiellement abdominale, le lymphome lymphoblastique de localisation médiastinale prédominante et le lymphome anaplasique à grandes cellules. Ces tumeurs progressent vite. Le traitement est différent pour les 3 groupes histologiques et nécessite un diagnostic rapide et de certitude. Dans les localisations profondes abdominales et médiastinales, les biopsies radioguidées permettent le plus souvent de poser le diagnostic de lymphomes et de son type histologique [1, 13]. Dans une étude publiée par Garret et coll, le diagnostic histologique a été obtenu dans 77% des cas [13]. Pour le lymphome de Hodgkin, dans les localisations profondes abdominales et médiastinales, les biopsies radioguidées permettent le plus souvent de poser le diagnostic [1]. Le problème de diagnostic différentiel sur un matériel exigu se pose avec les thymomes [6].

*Les sarcomes des tissus mous:* 6 cas ont été diagnostiqués dans notre série soit 7,7% des cas. C'est un groupe hétérogène de tumeurs malignes qui se développent à partir du mesoderme. Ils représentent 8 à 10% des tumeurs malignes de l'enfant et de l'adulte jeune [14]. Le rhabdomyosarcome représente environ la moitié des sarcomes de l'enfant [14]. Les autres sarcomes rencontrés chez l'enfant sont le groupe de PNET/sarcome d'Ewing, le synovialosarcome, le sarcome alvéolaire, le fibrosarcome infantile, le sarcome indifférencié et peu différencié et la tumeur desmoplastique à petites cellules rondes. La biopsie percutanée permet une chirurgie adaptée au type histologique ou la mise en œuvre d'un éventuel traitement néoadjuvant [9]. Les prélèvements percutanés sous imagerie sont actuellement un standard de prise en charge initiale des tumeurs conjonctives [15]. La démarche diagnostique des tumeurs des tissus mous de l'enfant est particulière car de nombreux sarcomes peuvent avoir un aspect histologique semblable [2]. Le pathologiste a souvent recours à l'immunohistochimie et à la biologie moléculaire pour poser le diagnostic.

*Le néphroblastome:* c'est une tumeur maligne embryonnaire développée à partir des cellules du blastème néphrogénique. Il représente environ 85% des tumeurs du rein de l'enfant [16]. L’âge moyen est de 36 mois chez les garçons et de 42 mois chez les filles. L'aspect histologique est classiquement celui d'un néphroblastome triphasique associant un contingent blastémateux, épithélial et mésenchymateux [16]. Des formes biphasiques et monophasiques sont possibles de même que des foyers de différenciation hétérologue [16]. Selon le protocole de la SIOP appliqué dans notre formation, les tumeurs rénales ne sont pas biopsiées et le diagnostic est radioclinique; elles sont traitées par une chimiothérapie préopératoire suivie par une néphrectomie chez les enfants âgés de plus de 6 mois [17].

*La tumeur myofibroblastique inflammatoire:* il s'agit d'une tumeur à malignité intermédiaire faite de d'une prolifération de cellules fusiformes associées à un infiltrat inflammatoire de lymphoplasmocytes et de polynucléaires [18]. C'est une tumeur de localisation ubiquitaire qui peut se voir chez l'enfant et l'adulte. Les récidives sont possibles et la transformation maligne est rare. Chez l'enfant, la localisation abdominale est plus fréquente que la localisation pulmonaire [18]. Sur le plan immunohistochimique, les cellules tumorales expriment l'anticorps anti Actine muscle lisse et l'ALK dans 50% des cas.

*L'hépatoblastome:* les tumeurs malignes du foie représentent environ 1% des tumeurs malignes de l'enfant dont les 2/3 correspondent à l'hépatoblastome [19]. Les garçons sont plus touchés que les filles. L’âge moyen au moment du diagnostic est de 18 mois. Histologiquement, il rappelle le foie embryonnaire et fœtal [19]. La biopsie percutanée peut être utilisée pour établir le diagnostic d'hépatoblastome [2]. Les autres diagnostics retrouvés dans notre série sont le sarcome d'Ewing (3 cas), la leucémie aigue (1 cas) et l'ostéosarcome (1 cas).

### Particularités de la biopsie radioguidée selon l'organe

*Les lésions hépatiques:* la biopsie radioguidée peut jouer un rôle essentiel dans le diagnostic des lésions hépatiques de l'enfant, quelles soit focales ou diffuses [4]. La biopsie percutanée est anodine chez les enfants ayant un bilan normal de la crase sanguine. Le doppler couleur est utilisé afin d’éviter les gros vaisseaux. Elle permet d'instaurer une chimiothérapie néoadjuvante pour les tumeurs chimiosensibles. Elle permet le diagnostic des hépatoblastomes et des carcinomes hépatocellulaires. En cas de contre indications, la biopsie peut être faite par voie transjugulaire [4].

*Les lésions pulmonaires:* la biopsie radioguidée doit être considérée comme le moyen diagnostic de première ligne dans la pathologie pulmonaire [5]. Les biopsies peuvent être échoguidées pour les lésions pleurales et pulmonaires superficielles, mais le plus souvent on a recours au scanner [20].

## Conclusion

Dans notre série, toutes localisations et tous diagnostics confondus, le diagnostic histopathologique était posé dans 89% des cas. La tumeur la plus fréquente était le neuroblastome suivi des lymphomes non hodgkiniens. Dans 10% des cas, la biopsie était non concluante pour les raisons suivantes: matériel non représentatif, matériel tumoral mais peu représentatif, immunomarquage non contributif. Cette technique nécessite une étroite collaboration entre clinicien, radiologue et pathologiste pour discuter son indication à fin de diminuer le nombre de biopsies peu ou non représentatives.

## References

[1] Hoffer FA (2005). Interventional radiology in pediatric oncology. Eur J Radiol..

[2] Garrett KM, Fuller CE, Santana VM, Shochat SJ, Hoffer FA (2005). Percutaneous Biopsy of Pediatric Solid Tumors. Cancer..

[3] Skoldenberg EG, Jakobson A, Elvin A, Sandstedt B, Olsen L, Christofferson RH (2002). Diagnosing childhood tumors: a review of 147 cutting needle biopsies in 110 children. J Pediatr surg..

[4] Bittles MA, Hoffer FA (2007). Interventional radiology and the care of the pediatric oncology patient. Surg Oncol..

[5] Cahill AM, Baskin KM, Kaye RD, Fitz CR, Towbin RB (2004). CT guided percutaneous lung biopsy in children. J Vasc Interv Radiol..

[6] Guimaraes AC, Chapchap P, Camargo B, Chojniak R (2003). Computed Tomography-Guided Needle Biopsies in Pediatric Oncology. J Pediatr Surg..

[7] Hussain HK, Kingston JE, Domizio P, Norton AJ, Reznek RH (2001). Imaging-guided core biopsy for the diagnosis of malignant tumors in pediatric patients. AJR Am J Roentgenol..

[8] Iezzoni JC, Fechner RE (1995). The biopsy: the pathologist's point of view. Surg Oncol Clin N Am..

[9] Ranchère-Vince D (2010). Tumeurs des parties molles de l'appareil locomoteur: que faire de la biopsie et de la pièce opératoire ‘ Rôle respectif du préleveur et du pathologiste. Arch Pediatr..

[10] Hugosson CO, Nyman RS, Cappelen-Smith JM, Akhtar M, Hugosson C (1999). Ultrasound-guided biopsy of abdominal and pelvic lesions in children: a comparison between fine-needle aspiration and 1,2 mm-needle core biopsy. Pediatr Radiol..

[11] Hassan SF, Mathur S, Magliaro TJ, Larimer EL, Ferrell LB, Vasudevan SA, Patterson DM, Louis CU, Russell HV, Nuchtern JG, Kim ES (2012). Needle core vs open biopsy for diagnosis of intermediate- and high-risk neuroblastoma in children. J Pediatr Surg..

[12] Frew JA, Lewis J, Lucraft HH (2013). The management of children with lymphomas. Clin Oncol (R Coll Radiol)..

[13] Garrett KM, Hoffer FA, Behm FG, Gow KW, Hudson MM, Sandlund JT (2002). Interventional radiology techniques for the diagnosis of lymphoma or leukemia. Pediatr Radiol..

[14] Merchant MS, Mackall CL (2009). Current Approach to Pediatric Soft Tissue Sarcomas. Oncologist..

[15] Shin HJ, Amaral JG, Armstrong D, Chait PG, Temple MJ, John P, Smith CR, Taylor G, Connolly BL (2007). Image-guided percutaneous biopsy of musculoskeletal lesions in children. Pediatr Radiol..

[16] Perlman E, Boccon-Gibod L (2004). Tumeurs du rein de l'enfant. Ann Pathol..

[17] Bhatnagar S (2009). Management of Wilms tumor: NWTS vs SIOP. J Indian assoc Pediatr Surg..

[18] Mergan F, Jaubert F, Sauvat F, Hartmann O, Lortat-Jacob S, Révillon Y, Nihoul-Fékété C, Sarnacki S (2005). Inflammatory myofibroblastic tumor in children: clinical review with anaplastic lymphoma kinase, Epstein-Barr virus, and human herpesvirus 8 detection analysis. J Pediatr Surg..

[19] Litten JB, Tomlinson GE (2008). Liver Tumors in Children. Oncologist..

[20] Fontalvo LF, Amaral JG, Temple M, Chait PG, John P, Krishnamuthy G, Smith C, Connolly B (2006). Percutaneous US-guided biopsies of peripheral pulmonary lesions in children. Pediatr Radiol..

